# Copper–zinc imbalance induces kidney tubule damage and oxidative stress in a population exposed to chronic environmental cadmium

**DOI:** 10.1007/s00420-019-01490-9

**Published:** 2019-11-15

**Authors:** Sang-Yong Eom, Dong-Hyuk Yim, Mingai Huang, Choong-Hee Park, Guen-Bae Kim, Seung-Do Yu, Byung-Sun Choi, Jung-Duck Park, Yong-Dae Kim, Heon Kim

**Affiliations:** 1 Department of Preventive Medicine, College of Medicine, Chungbuk National University, 1 Chungdae-ro, Seowon-gu, Cheongju, 28644 Republic of Korea; 2 Office of Public Healthcare Service, Chungbuk National University Hospital, Cheongju, Republic of Korea; 3 Department of Preventive Medicine, Chung-Ang University College of Medicine, Seoul, Republic of Korea; 4 Environmental Health Research Division, Environmental Health Research Department, National Institute of Environmental Research, Incheon, Republic of Korea

**Keywords:** Cadmium, Essential metal, Environmental exposure, Kidney tubule damage, Oxidative stress

## Abstract

**Purpose:**

This cross-sectional study aimed to assess the effect of environmental cadmium (Cd) exposure and essential metal imbalance on renal tubular damage and oxidative stress in 979 adults living in a Cd-polluted area near an abandoned copper (Cu) refinery.

**Methods:**

We analyzed urinary Cd concentrations, renal tubular damage and oxidative stress markers, such as beta-2 microglobulin (β2-MG) and *N*-acetyl-β-d-glucosaminidase (NAG) activity and urine malondialdehyde (MDA) levels. The serum copper-to-zinc ratio (CZR) was used as an essential metal imbalance indicator. We divided the subjects into two Cd exposure groups based on the reference level of urinary Cd for renal dysfunction (2 μg/g creatinine).

**Results:**

The geometric mean concentration of urinary Cd in all subjects was 2.25 μg/g creatinine. In both low and high Cd exposure groups, urinary Cd levels were positively correlated with urinary NAG activity, but not with serum CZR. After multivariate adjustment, serum CZR was strongly associated with urinary β2-MG levels in the low Cd exposure group (*β* = 1.360, *P* = 0.019) and was significantly associated with urinary MDA levels, regardless of Cd exposure level. In addition, the risk of renal tubular damage was significantly associated with urinary Cd level, particularly in the lowest or highest CZR tertile groups.

**Conclusions:**

Essential metal imbalance may be a determinant of oxidative stress and renal tubular damage in a chronically Cd-exposed population, and proper zinc supplementation will be effective in preventing adverse health effects due to Cd exposure.

## Introduction

Cadmium (Cd) is widely distributed in the environment and is a major environmental pollutant that threatens human health. Under normal conditions, absorbed Cd is filtered through the glomerulus, reabsorbed in the proximal tubules, and accumulated in the kidneys (Jarup et al. 1998). In individuals with a chronic exposure to Cd, approximately 50% of the absorbed Cd is distributed in the kidneys and causes injury to renal microtubules. When kidney tubules are injured, urinary excretions of *N*-acetyl-d-glucosaminidase (NAG) and beta-2 microglobulin (β2-MG) increase (Bernard 2008; Jarup et al. 1998).

The gastrointestinal absorption of Cd is affected by the nutritional status of individuals, such as the body levels of iron (Fe) and zinc (Zn) (EFSA 2009; Ryu et al. 2004), and Cd interacts metabolically with some essential metals such as Zn, copper (Cu), Fe, and calcium (Ca) (Goyer 1997). Those metal ions and Cd are competitively transported by common transporters [i.e., divalent metal transporter 1, Zrt Irt-related protein 8 (ZIP8), and ZIP14] (Jenkitkasemwong et al. 2012; Vesey 2010) and tightly bound to metallothionein (MT) in the systemic circulation. Therefore, the concentration of essential metals is affected by the concentration of Cd in the body.

Cd induces reactive oxygen species, which may play a role in acute and chronic Cd toxicity (Satarug et al. 2017; Shaikh et al. 1999; Thevenod and Friedmann 1999), while Cu and Zn are antioxidant trace elements because they act as the cofactors of cytoplasmic superoxide dismutase (Pokusa and Kralova Trancikova 2017). A recent study reported that an imbalance between Zn and Cu is associated with renal dysfunction in humans, which is mediated by oxidative stress (Hamasaki et al. 2016).

As the Cd concentration in the body increases, Cu and Zn levels can change and lead to an imbalance of the Cu-to-Zn ratio (CZR). Cd can simultaneously cause renal tubular damage as well as an imbalance of the CZR. This study aimed to test whether a CZR imbalance is an intervening factor in the toxic mechanism of Cd on renal tubules or an independent risk factor for renal tubular damage.

## Materials and methods

### Study participants

This cross-sectional study was conducted in a Cd-contaminated area near the Janghang Copper Refinery, which was closed in 1989. The Janghang Copper Refinery is located in the Seocheon-Gun, Chungnam Province, on the west coast of the Korean Peninsula. It is reported that, within a 7-km radius of the refinery, soil Cd levels range from 1.6 to 27.2 mg/kg and decrease with increasing distance from the refinery (Kim and Chon 1993).

The selection of study participants was described in detail in previous studies (Kim et al. 2014, 2016). In brief, the study included 985 adults aged ≥ 30 years who had been living within 15 km of the closed refinery. All subjects were provided with information about the purpose of this study, and they provided written consent. Experienced interviewers directly interviewed the subjects with a questionnaire that included demographic and lifestyle information, including dietary habits, smoking habits (current smoking status, average number of cigarettes smoked daily, total duration of smoking), alcohol consumption, occupation, duration of current residence, and past medical history. Non-smokers were defined as individuals who had never smoked cigarettes or who had smoked fewer than 100 cigarettes in their lifetimes. Whole blood and spot urine samples were collected from the participants and stored at − 80 °C until analysis. The participants with minimal or no urine (*n* = 6) were excluded from this study. Finally, a total of 979 participants were included.

### Determination of trace metal levels in biological samples

We quantified the concentrations of Cd in the urine and Cu and Zn in the serum of subjects. The determination of Cd in urine was performed with a flameless atomic absorption spectrophotometer (Model Z-8270, Hitachi) equipped with a Zeeman graphite furnace. Briefly, urine was added to nitric acid and diluted with di-ammonium hydrogen phosphate and 1% Triton X-100, followed by vigorous mixing. The detection limit was 0.01 µg/L for Cd in urine. Cu and Zn in serum were measured using inductively coupled plasma mass spectrometry (Elan DRC-e, Perkin Elmer, USA) at a radio frequency power of 1550 W. The argon plasma gas flow rate and argon carrier gas flow rate were 15 L/min and 1.04 L/min, respectively. The kinetic energy discrimination mode using helium gas (4.3 μL/min) was applied to measure Cu and Zn. The detection limits were 0.54 and 0.15 µg/dL for serum Cu and Zn, respectively. There were no samples with concentrations of any element below the detection limits.

### Determination of NAG activity, β2-MG, and malondialdehyde (MDA) in the urine

As markers for renal tubular damage, urinary NAG activity and β2-MG levels were measured.

Urinary NAG activity was quantified using a commercial kit (Shionogi, Osaka, Japan) according to the manufacturer’s protocol. In brief, a synthetic substrate solution (1 μL) was incubated at 37 °C for 5 min. After centrifugation of the urine samples, 50 μL of the supernatant was mixed with a warm synthetic substrate solution and then incubated in a 37 °C water bath for 15 min. The stopping solution (2 μL) was added, and the absorbance of the samples and NAG standard solution were measured at 580 nm using a spectrophotometer. The urinary β2-MG level was measured using a commercial kit (Enzygnost β2-MG Micro Kit; Behring Institute, Mannheim, Germany) according to the manufacturers’ instructions. The test kit principle was based on the solid phase enzyme-linked immunosorbent assay. The assay system utilized a monoclonal anti-β2-MG antibody for solid phase immobilization and an anti-β2-MG-horseradish peroxidase conjugate solution.

As a marker of oxidative stress, the concentration of MDA in urine was determined by measuring the level of thiobarbituric acid reactive substances (TBARS) using a high-performance liquid chromatographic (HPLC) system with a fluorescence detector (Agarwal and Chase 2002). Briefly, 50 μL of 0.05% butylated hydroxytoluene (B1378, Sigma-Aldrich, St. Louis, MO, USA), 150 μL of 0.1125 N nitric acid (438073, Sigma-Aldrich), and 150 μL of 42 mM thiobarbituric acid (TBA, T5500, Sigma-Aldrich) were added to a 50 μL aliquot of urine sample or 50 μL of 1,1,3,3-tetramethoxypropane (Alfa Aesar, Heysham, UK) standard solution and vortex-mixed. The samples were then heated at 100 °C for 1 h, and 300 μL of *n*-butanol (W217816, Sigma-Aldrich) was added for the extraction of TBARS. After centrifugation, 10 µl of the supernatant was injected into the HPLC system, which consisted of a pump (Lsp 930; Younglin, Seoul, Korea), an automatic injector (SIL 10Avp; Shimadzu, Kyoto, Japan), a fluorescence detector (RF-10AxL; Shimadzu), and a data acquisition module (Autochro-200; Younglin). The column was a 150 mm long reverse-phase column (TSK-GEL ODS-80TM; Tosoh), and the mobile phase was potassium dihydrogen phosphate:methanol:acetonitrile (60:25:15, v/v/v) at a flow rate of 1 μL/min. The excitation/emission wavelengths were 515/553 nm. The limit of detection of MDA in the urine was 0.07 μmol/L, and the intra-assay coefficient of variation for the pooled urine sample was 5.25%.

### Statistical analysis

All data on the levels of trace metals were log-transformed since the distribution was right skewed. Participants were assigned to exposure groups based on the Cd level in urine being low (< 2 μg/g creatinine) or high (≥ 2 μg/g creatinine), as per previously reported thresholds (Buchet et al. 1990). Differences in the demographic or lifestyle factors of the groups were compared using the Chi-square test. Statistical comparisons of the means of various biomarkers were performed using Student’s *t* test. The Pearson’s correlations between the log-transformed levels of Cd, Cu and Zn and the other biomarkers were evaluated. Multiple linear regression models were used to test the associations of the serum CZR and the urinary Cd level with renal tubular damage or oxidative stress. Each model included age, sex, body mass index, smoking status, drinking status, diabetes, hypertension and urinary creatinine concentration as covariates. In the regression analysis, we used creatinine-unadjusted values for urinary biomarkers and included urinary creatinine value in the models as covariate to adjust urine dilution. The association of urinary Cd levels with renal tubular damage, according to serum CZR levels, was evaluated by multivariate logistic regression analyses. The serum CZR level was categorized into tertiles (lowest, middle, and highest groups). ‘‘High NAG’’ and ‘‘high β2-MG’’ were defined as greater than 11.5 unit/g creatinine and 300 µg/g creatinine, respectively, which are critical reported values for renal tubular damage (Bernard 2008). All statistical analyses were performed using the Statistical Package for the Social Sciences (SPSS) software version 24.0 (IBM, Armonk, NY, USA).

## Results

The general characteristics of the 979 study participants are presented in Table 1. The geometric mean concentration of Cd in urine was 2.25 μg/g creatinine. There were 396 subjects (40.4%) with urinary Cd values < 2.0 μg/g creatinine (low Cd exposure group) and 583 subjects (59.6%) with urinary Cd values ≥ 2.0 μg/g creatinine (high Cd exposure group). The high Cd exposure group had more elderly and female subjects than the low Cd exposure group. Meanwhile, the low Cd exposure group had more current- or ex-smokers and more alcohol drinkers than the high Cd exposure group. There were no between group differences for the prevalence of diabetes, hypertension, or body mass index. Serum Cu and Zn levels and urinary NAG and MDA were significantly higher in the high Cd exposure group than in the low Cd exposure group. However, serum CZR and urinary β2-MG were not significantly different between the two groups.

**Table 1:** General characteristics of the study participants

Variables	Total	Cadmium exposure		*P* value^a^
		Low (< 2 μg/g creatinine)	High (≥ 2 μg/g creatinine)	
Total, *N* (%)	979 (100.0)	396 (40.4)	583 (59.6)	–
Age, years, mean ± SD	64.33 ± 11.50	61.67 ± 13.65	66.14 ± 9.66	< 0.001
> 65 years, *N* (%)	550 (56.2)	194 (49.0)	356 (61.1)	< 0.001
> 75 years, *N* (%)	172 (17.6)	63 (15.9)	109 (18.7)	0.307
Females, *N* (%)	578 (59.0)	153 (38.6)	425 (72.9)	< 0.001
Current or ex-smokers, *N* (%)	309 (32.0)	161 (41.3)	148 (25.7)	< 0.001
Alcoholic drinkers, *N* (%)	482 (49.4)	234 (59.4)	248 (42.6)	< 0.001
Diabetes, *N* (%)	180 (18.4)	62 (15.7)	118 (20.2)	0.069
Hypertension, *N* (%)	636 (65.0)	256 (64.8)	380 (65.2)	0.912
Body mass index, kg/m^2^, mean ± SD	24.57 ± 3.50	24.71 ± 3.52	24.47 ± 3.49	0.266
Urinary cadmium, μg/g creatinine^b^	2.25 (2.14, 2.34)	1.10 (1.05, 1.15)	3.63 (3.51, 3.76)	< 0.001
Serum copper, μg/dL^b^	91.84 (89.12, 93.69)	86.03 (82.85, 89.33)	95.33 (92.67, 98.06)	< 0.001
Serum zinc, μg/dL^b^	76.71 (75.19, 78.26)	72.66 (70.28, 75.13)	79.04 (77.12, 81.00)	< 0.001
Serum copper-to-zinc ratio^b^	1.20 (1.17, 1.22)	1.18 (1.16, 1.21)	1.21 (1.18, 1.23)	0.251
Urinary NAG, unit/g creatinine^b^	2.83 (2.56, 3.10)	2.16 (1.84, 2.53)	3.35 (2.98, 3.78)	< 0.001
Urinary β_2_-MG, μg/g creatinine^b^	23.10 (19.49, 27.11)	17.92 (13.80, 23.27)	24.31 (19.89, 29.71)	0.066
Urinary MDA, μmol/g creatinine^b^	1.67 (1.62, 1.72)	1.58 (1.51, 1.65)	1.73 (1.66, 1.80)	0.002

*SD* standard deviation, *NAG N*-acetyl-β-d-glucosaminidase, *β*_*2*_*-MG* β_2_-microglobulin, *MDA* malondialdehyde

^a^The *P* values were determined by *t* tests or Chi-squared tests for the difference between the low and high cadmium exposure groups

^b^Values are presented as geometric means with 95% confidence intervals

Urinary Cd levels were positively correlated with the Cu and Zn levels in serum, but not with CZR in both the low and high Cd exposure groups. Urinary NAG activity and MDA levels were significantly correlated with urinary Cd in both Cd exposure groups. Serum CZR was significantly correlated with urinary MDA levels, but not with the urinary NAG activity. In the low Cd exposure group, serum CZR was positively correlated with the urinary β2-MG level (Table 2).

**Table 2:** Pearson’s correlation coefficients among the biomarkers of trace elements, renal tubular damage, and oxidative stress

Cadmium exposure group	Variables^a^	Serum copper, μg/L	Serum zinc, μg/L	Serum copper-to-zinc ratio	Urinary NAG, unit/g creatinine	Urinary β_2_-MG, µg/g creatinine	Urinary MDA, µmol/g creatinine
Total	Urinary Cd, μg/g creatinine	0.191**	0.185**	0.043	0.200**	0.076*	0.118**
	Serum copper, μg/dL		0.744**	0.511**	0.168**	0.038	0.038
	Serum zinc, μg/dL			− 0.194**	0.157**	0.002	− 0.082*
	Serum copper-to-zinc ratio				0.046	0.054	0.161**
	Urinary NAG, unit/g creatinine					0.355**	0.015
	Urinary β_2_-MG, µg/g creatinine						0.096**
Low (< 2 μg/g creatinine)	Urinary Cd, μg/g creatinine	0.182**	0.188**	0.024	0.151**	− 0.084	0.066
	Serum copper, μg/dL		0.779**	0.485**	0.261**	0.062	0.047
	Serum zinc, μg/dL			− 0.170**	0.257**	− 0.020	− 0.067
	Serum copper-to-zinc ratio				0.054	0.125*	0.165**
	Urinary NAG, unit/g creatinine					0.340**	− 0.072
	Urinary β_2_-MG, µg/g creatinine						0.085
High (≥ 2 μg/g creatinine)	Urinary Cd, μg/g creatinine	0.103*	0.104*	0.017	0.142**	0.160**	0.071
	Serum copper, μg/dL		0.705**	0.533**	0.064	0.006	0.012
	Serum zinc, μg/dL			− 0.225**	0.048	0.006	− 0.115**
	Serum copper-to-zinc ratio				0.031	0.001	0.154**
	Urinary NAG, unit/g creatinine					0.360**	0.052
	Urinary β_2_-MG, µg/g creatinine						0.094**

*Cd* cadmium, *NAG N*-acetyl-β-d-glucosaminidase, *β*_*2*_*-MG* β_2_-microglobulin, *MDA* malondialdehyde

^a^All variables are log-transformed

*P < 0.05, **P < 0.01

In the multivariate analysis—adjusted for potential confounding factors such as age, sex, body mass index, smoking, alcohol consumption, diabetes, hypertension, and urinary creatinine concentration—urinary Cd levels were significantly associated with urinary NAG activity and β2-MG levels in the high Cd exposure groups. Furthermore, there was a significant and positive association between the serum CZR and urinary MDA was observed in both Cd exposure groups. However, serum CZR was significantly associated with urinary β2-MG levels only in the low Cd exposure group (Table 3).

**Table 3:** Multiple linear regression analysis for renal tubular damage and oxidative stress

Dependent variables	Independent variables	Cadmium exposure group			
		Low (< 2 μg/g creatinine)		High (≥ 2 μg/g creatinine)	
		*β*^a^	*P* value	*β*^a^	*P* value
Urinary NAG (unit/L)	Urinary Cd (μg/L)	0.271	0.128	0.482	0.002
	Serum copper-to-zinc ratio	0.357	0.284	0.091	0.710
Urinary β_2_-MG (μg/L)	Urinary Cd (μg/L)	− 0.409	0.184	0.703	0.006
	Serum copper-to-zinc ratio	1.360	0.019	0.120	0.771
Urinary MDA (µmol/L)	Urinary Cd (μg/L)	0.051	0.276	− 0.027	0.549
	Serum copper-to-zinc ratio	0.243	0.006	0.265	< 0.001

*Cd* cadmium, *NAG N*-acetyl-β-d-glucosaminidase, *β*_*2*_*-MG* β_2_-microglobulin, *MDA* malondialdehyde

^a^Multiple linear regression adjusted for age, sex, body mass index, smoking status, alcoholic drinking status, diabetes, hypertension and urinary creatinine concentration. Both independent and dependent variables are log-transformed

We evaluated associations between urinary Cd level and the risk of renal tubular damage according to serum CZR levels were evaluated (Table 4). In high Cd exposure group, the prevalence of high NAG activity increased with the serum CZR tertile levels. Among the highest serum CZR tertile group, the risk of high NAG activity was 4.98 times higher for the high Cd exposure group than for the low Cd exposure group. After controlling for various potential confounders, statistical significance remained (adjusted odds ratios = 3.76, 95% CI 1.24–11.44). In both lowest and highest serum CZR tertile group, the risk of high β2-MG levels was significantly higher in the high Cd exposure group than in the low Cd exposure group. However, there was no similar association observed within the middle serum CZR tertile group.

**Table 4:** Associations of urinary Cd levels with renal tubular damage according to serum copper-to-zinc ratio

Level of CZR	High NAG (> 11.5 unit/g creatinine)				High β_2_-MG (> 300 µg/g creatinine)			
	*N* (%)		OR (95% CI)^a^		N (%)		OR (95% CI) ^a^	
	Low Cd^b^	High Cd	Crude	Adjusted^c^	Low Cd^b^	High Cd	Crude	Adjusted^c^
Tertile 1 (CZR < 1.1)	3 (2.31)	15 (7.98)	3.67 (1.04, 12.95)	3.37 (0.84, 13.51)	3 (2.31)	19 (10.11)	4.76 (1.38, 16.43)	4.22 (1.09, 16.36)
Tertile 2 (1.1 ≤ CZR < 1.3)	10 (7.09)	24 (12.24)	1.83 (0.85, 3.96)	1.31 (0.57, 3.01)	12 (8.51)	15 (7.65)	0.89 (0.40, 1.97)	0.79 (0.33, 1.85)
Tertile 3 (CZR ≥ 1.3)	4 (3.05)	27 (13.57)	4.98 (1.70, 14.60)	3.76 (1.24, 11.44)	4 (3.05)	19 (9.55)	3.35 (1.11, 10.08)	3.26 (0.99, 10.74)

*Cd* cadmium, *NAG N*-acetyl-β-d-glucosaminidase, *β*_*2*_*-MG* β_2_-microglobulin, *CZR* serum copper-to-zinc ratio, *OR* odds ratios, *CI* confidence interval

^a^OR (95% CI) for renal tubular damage, comparing the low Cd exposure and the high Cd exposure group by level of CZR tertiles

^b^Cadmium exposure was divided into a low (urinary Cd < 2 μg/g creatinine) or high (urinary Cd ≥ 2 μg/g creatinine) exposure group

^c^Adjusted for age, sex, body mass index, smoking status, alcoholic drinking status, diabetes, and hypertension

## Discussion

The present study evaluated whether an imbalance in the levels of Zn and Cu is associated with renal tubular damage or oxidative stress in a population living in a Cd-polluted area. Serum CZR was found to be a determinant of oxidative stress and was significantly associated with microscopic damage to the proximal tubules in the low and chronically Cd-exposed population. In addition, it was found that the imbalance between Cu and Zn could exacerbate the risk of kidney damage due to Cd.

In this study, we divided subjects into Cd exposure groups based on a threshold level of 2 μg/g creatinine. This is believed to be the level that can cause Cd-induced renal tubular damage (Buchet et al. 1990; Prozialeck and Edwards 2010). The urinary Cd level among the low Cd exposure group in our study (GM 1.10, 95% CI 1.05–1.15 μg/g creatinine) was comparable to levels observed in the general Korean adult population (GM range 0.95–1.36 μg/g creatinine) (Eom et al. 2017; Huang et al. 2013; Lim et al. 2016). However, the mean urinary Cd level in the high Cd exposure group (GM 3.63, 95% CI 3.51–3.76 μg/g creatinine) was more than twice of that reported in previous studies on general population (Eom et al. 2017; Huang et al. 2013; Lim et al. 2016). Compared to the low Cd exposure group, the high Cd exposure group had many more women and individuals classified as elderly, both of which are known predictors of an elevated body burden of Cd (Jarup et al. 1998; Vacchi-Suzzi et al. 2016). In addition, the low Cd exposure group had significantly more current- or ex-smokers than the high Cd exposure group. This is due to the confounding effect caused by the high proportion of women who were non-smokers in the high Cd exposure group. In fact, in the stratification analysis by sex, the proportion of ex-smokers was higher in the high-Cd exposed group than in the low-exposed group for both men and women (data not shown).

In our study, the serum Cu and Zn levels of the high Cd exposure group were significantly higher than those of the low Cd exposure group and were positively correlated with the levels of Cd in urine. This result can be explained by the simultaneous exposure to Cd, Cu, and Zn, which are the main products or byproducts of the Cu smelter. Similarly, in a previous study with copper smelter workers, positive correlations between Cd, Cu, and Zn in tissues were reported (Gerhardsson et al. 2002). However, it was reported that serum Zn levels are inversely associated with blood Cd concentrations in American adults (Vance and Chun 2015), and chronic low-level Cd exposure was associated with reduced Cu and Zn reabsorption (Satarug et al. 2018).

An imbalance in the Cu/Zn level had different effects on renal tubular damage, particularly β2-MG, depending on the Cd exposure level. In the high Cd exposure group, Cd was an independent risk factor for renal tubular damage, and the same results were found in numerous previous studies (Bernard 2004, 2008; Eom et al. 2017; Jarup et al. 1998; Satarug et al. 2010). However, in the high Cd exposure group, the CZR effect on β2-MGuria was relatively weaker than that of Cd. Interestingly, in the low Cd exposure group, the urinary β2-MG concentration was positively associated with the serum CZR but not with the urinary Cd level. Considering that the urinary Cd level was not correlated with the CZR, these results suggest that a Cu/Zn imbalance may be an independent factor for the elevated urinary β2-MG level in the low Cd exposure group. Similarly, previous studies have reported that an overload or deficiency of Cu or Zn is associated with renal dysfunction (Hamasaki et al. 2016; Ikeda et al. 2007; Liu et al. 2018). In particular, Ikeda et al. (2007) found that β2-MG levels are more related to Cu levels than to Cd levels in women without environmental Cd exposure (Ikeda et al. 2007). Schantz et al. reported that an induction of MT by zinc acts as a potential endogenous antioxidant in renal proximal tubular cells, which has a protective effect against the reduction of kidney function (Schanz et al. 2017). Therefore, this study suggests that the balance of essential metals, such as Cu and Zn, plays an important role in renal dysfunction, especially in groups with low levels of Cd exposure. The CZR is a more important indicator than each concentration of these trace metals for evaluating trace metal imbalance (Osredkar and Sustar 2011).

In the present study, the urinary NAG activity was not associated with the CZR but with the urinary Cd levels. In the high exposure group, the urinary β2-MG levels were significantly correlated with the urinary Cd levels. Urinary NAG activity reflects ongoing damage in the tubular epithelial cells, while the β2-MG level reflects a decreased tubular reabsorption capacity (Bernard 2008; Fassett et al. 2011). When the CZR was stratified, the risk of both NAG and β2-MG abnormalities caused by Cd showed a U-type pattern with relatively increased CZR tertile levels. Although these results suggest that CZR has a mechanism of renal toxicity different from that of Cd, it suggests that the kidney damage caused by Cd can be controlled by balancing essential metals.

The mechanism of linking Cd exposure and renal tubular damage has not yet been fully understood. Cd-induced oxidative stress may play an important role in renal damage (Prozialeck and Edwards 2010; Thevenod 2003). In this study, the serum CZR was significantly associated with the MDA levels in urine in both Cd exposure groups after adjusting for potential confounding factors. In contrast, the urinary Cd was not associated with the level of urinary MDA. This finding suggests that although Cd can cause a decrease in renal Zn reabsorption, which leads to Cu and Zn imbalances (Satarug et al. 2018), the CZR imbalance is an independent risk factor for renal tubular damage by inducing oxidative stress. Similar to our findings, several studies have shown a correlation between the imbalance in trace elements and oxidative stress (Guo and Wang 2013; Lin et al. 2014; Ozturk et al. 2013). Previous studies have also shown that Zn supplementation increases the antioxidant enzyme activity associated with Zn and reduces oxidative stress and inflammatory responses (Kloubert and Rink 2015; Mariani et al. 2008).

Both serum Cu and Zn levels reflect dietary intake and supplementation (Hess et al. 2007). Imbalance between Cu and Zn is often caused by inadequate dietary factors (Lonnerdal 2000; Ma and Betts 2000) and influenced by physiological conditions (i.e., age, infection, and anemia), alcohol drinking, malabsorption due to gastrointestinal diseases, inflammatory conditions, and genetics (Osredkar and Sustar 2011; Roohani et al. 2013). However, this study was limited by its cross-sectional design and was therefore unable to identify the specific cause and prolonged period of Cu and Zn imbalance. In addition, the temporal relationship between the imbalance of essential element and renal tubular damage or oxidative stress remains unclear. Consequently, our results should be interpreted with caution.

In conclusion, CZR imbalance is a risk factor for renal tubular damage by inducing oxidative stress independent of Cd. The results of this study suggest that an imbalance between Cu and Zn must be evaluated to identify kidney damage due to a chronic exposure to heavy metals, and proper Zn supplementation will be effective in preventing adverse health effects due to Cd exposure.
